# Correspondence

**Published:** 1895-02

**Authors:** Isaiah Michener

**Affiliations:** Carversville, Pa.


### CORRESPONDENCE.

CARVERSVILLE, PA., JANUARY 31, 1895.

*Editor of Journal of Comparative Med. and Veter. Archives.*

THE editorial, "Roads to Veterinary Education," is an excellent article. I am at a loss to know in what way the professors of colleges should proceed or adopt plans to ascertain whether the persons offering themselves as students have the natural ability and love for animals to insure their taking a proper interest in the science. A person to become a good veterinarian should have a philosophical mind, so that he could trace causes up to their effects and effects back to their causes. To do that he must have large reasoning faculties; his perceptive faculties should be well developed, so as to take notice of every symptom and movement that a sick animal will make; his organ of comparison should be large, to enable him to compare symptoms that simulate each other in different diseases; his mechanical talent should be good, for he will find use for it every day in practice; in fact, he should have a well-developed brain, or you will fail to make a good veterinary surgeon out of him. You cannot make a good wagon wheel out of a pine board, although you have painted, polished, and varnished it so that it will look as well as any wheel made of the best material. Now, what is the wheel good for? The answer is nothing. So it is with him that has no talent for the profession. He may learn to recite and answer questions, and still be of no more account when he goes into practice than the good-for-nothing wheel is on the road. I frequently tell my neighbor doctors that any kind of a stick will do to make a human doctor out of, because their patients tell them what is the matter, and they have nothing to do but to prescribe.

I had at one time two young men, well educated, to call on me, saying they wanted to read veterinary medicine. I took them in; they read books attentively and went with me frequently, and when I was examining a horse and asking questions to get the history of the case they would be walking around, looking at something else. A nearby farmer had a horse taken (it was supposed) early in the evening with enteritis. In the morning, when called upon, I took them with me. I

soon saw the disease had done its work, and the mare would die. I left them with instructions to watch every symptom and movement that the patient made until she died, and then we would make a post-mortem, but in half an hour after I left they came home. I asked why they had left so soon. They replied they had seen how the horse acted, and that was enough. I advised them to quit reading veterinary medicine, which they did.

I write this to show that many men who have no talent for the profession will attend lectures and obtain diplomas. To remedy this evil do not graduate anyone under a three-year course of twenty-four weeks in college, and the intervals spent with a good practitioner, where he can be getting a practical knowledge every day. I look upon a man with a diploma like a stallion with a registered pedigree; if he be individually worthless, the diploma and the pedigree are of no account.

I see by THE JOURNAL that the intelligence manifested by horses and dogs is claiming attention. If I do not forget, I will give you some of my experience on that line, and I may be foolish enough to offer a reason for "Mazeppa's" performance; but I do not expect to do anything more of any account, because I feel that I am on the down grade to the last depot, running steady and easy, expecting to arrive there when the law of Nature for the outlet of human life has been fulfilled, which is worn-out old age. Perhaps you may find a grain of wheat in all this chaff.

Very truly yours,

ISAIAH MICHENER.

---

No less that four bills are pressing for consideration at the present session of the State Legislature in Pennsylvania, looking to a better and more comprehensive method of dealing with contagious and infectious diseases of live stock. Active, aggressive workers are vigorously pushing them all, and many prophesies are in the air as to the outcome. A department of agriculture, with a cabinet position for the head in the Governor's official family, with sections devoted to animal industry, forestry, and ornithology, under its supervision. The creation of a State veterinarian, as an official of the State Government, is being considered.

